# Post-TAVR Conduction Disturbances: Risk Stratification Using Consensus Algorithm and Membranous Septum Length

**DOI:** 10.1016/j.jacasi.2026.05.021

**Published:** 2026-07-03

**Authors:** Yuta Kobayashi, Yusuke Enta, Masaki Nakashima, Makoto Saigan, Natsuko Satomi, Yung Teng, Daishi Tazawa, Yoshiko Munehisa, Yukihiro Hayatsu, Norio Tada

**Affiliations:** a Department of Cardiology, Sendai Kousei Hospital, Sendai, Japan; b Department of Cardiovascular Medicine, Faculty of Medicine and Graduate School of Medicine, Hokkaido University, Sapporo, Japan; c Department of Cardiovascular Surgery, Sendai Kousei Hospital, Sendai, Japan

**Keywords:** conduction disturbances, membrane septum, transcatheter aortic valve implantation

New-onset conduction disturbances (CDs) after transcatheter aortic valve replacement (TAVR) represent severe complications that can result in sudden cardiac death. A consensus algorithm was introduced in 2019 to balance the prevention of sudden cardiac death from CDs after TAVR with the goal of optimizing early discharge strategies.^1^ Although this algorithm has a high negative predictive value (NPV) for identifying patients unlikely to require permanent pacemaker implantation (PPMI), stratifying risk among high-risk patients remains challenging.^2^, ^3^, ^4^ Furthermore, because this algorithm relies only on electrocardiogram (ECG) changes, the role of anatomical features, such as membranous septum (MS) length, is not yet well understood. This study aimed to validate the CD consensus algorithm and determine whether the addition of anatomical parameters, particularly MS length, could improve risk stratification in high-risk patients.

This retrospective study included 586 consecutive patients with severe aortic stenosis who underwent TAVR between June 2022 and December 2024. The study protocol was approved by the ethics committee of Sendai Kousei Hospital. Patients who required emergent conversion to open-heart surgery (n = 3), valve-in-valve procedures (n = 20), or had pre-existing PPMs or implantable cardioverter-defibrillators (n = 22) were excluded. Finally, 541 patients were included in this study.

All standard 12-lead ECGs were recorded in the supine resting position at baseline, immediately after the procedure, and daily for 2 days post-TAVR. Computed tomography was performed using a 320-detector row Aquilion ONE VISION Edition computed tomography scanner (Toshiba Medical Systems). The MS length was measured in the coronal view using Ziostation2 software, version 2.9.3.0 (Ziosoft).

PPMs were implanted based on each attending cardiologist's judgment in cases of complete heart block (CHB) or advanced high-grade atrioventricular block (HAVB) caused by documented symptomatic bradycardia. Patients at high risk of developing advanced CDs who did not receive PPMI were followed up with ECG monitoring.

The primary endpoint of this study was the incidence of PPMI for HAVB or CHB within 30 days after TAVR, as assessed according to 3 algorithm-based management recommendations: eligibility for early discharge, higher risk of HAVB or CHB, and need for PPMI.

Forty-three (7.9%, 95% CI: 5.9%-10.6%) patients received PPMI within 30 days after TAVR. Of the total PPMs, 19% (8 of 43) were implanted on the first day, and 49% (21 of 43) were implanted within 4 days. Based on the presence or absence of preexisting and new ECG changes, group assignments were made immediately after TAVR according to the expert consensus algorithm: 234 patients (43%) were categorized into group 1, 56 (10%) into group 2, 66 (12%) into group 3, 145 (26%) into group 4, and 40 patients (7%) into group 5. The consensus algorithm resulted in the following management recommendations: early discharge for 388 patients (72%), classification as high risk for HAVB or CHB for 126 (23%) and PPMI for 27 (5%). The PPMI rates were 85.2% (23 of 27, 95% CI: 66.5%-94.3%) in the group in which PPMI was recommended, 11.1% (14 of 126, 95% CI: 6.7%-17.9%) in the group that was considered at high risk for HAVB or CHB, and 1.6% (6 of 388, 95% CI: 0.7%-3.4%) in the group that was recommended for early discharge. These correspond to NPVs of 14.8%, 88.9%, and 98.4%, respectively. The PPMI rates in high-risk patients, divided into 2 subgroups based on a median MS length of 2.8 mm, were 4.8% (3 of 63, 95% CI: 1.5%-13.9%) in those with an MS length ≥2.8 mm and 17.5% (11 of 63, 95% CI: 9.9%-29.0%) in those with an MS length <2.8 mm (Figure 1). The receiver operating characteristic analysis identified 2.8 mm as the optimal cutoff value of the MS length for predicting PPMI in patients at high risk for HAVB/CHB, with a sensitivity of 78.6% (95% CI: 49.2%-95.3%), specificity 53.6% (95% CI: 43.9%-63.0%), and c-index of 0.66 (95% CI: 0.54-0.78). Four patients (0.7%) died within 30 days of TAVR. Notably, none of the deaths were classified as sudden cardiac deaths caused by bradyarrhythmias.

**Figure 1:**
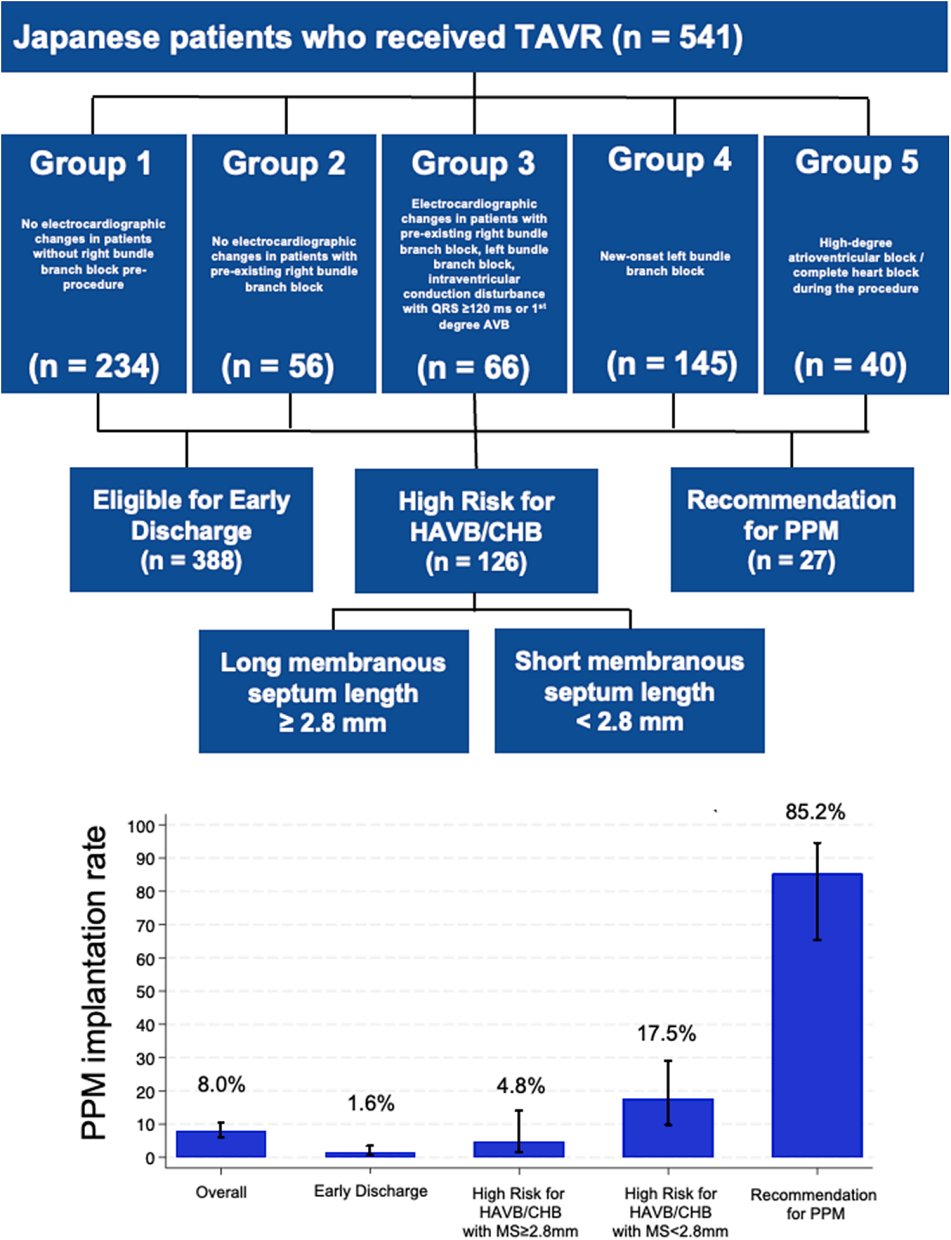
External Validation of the Expert Consensus Algorithm Incorporating MS Length Flow diagram of the present study and incidence of PPMI according to groups classified by the expert consensus algorithm incorporating MS length. AVB = atrioventricular block; CHB = complete heart block; HAVB = high-grade atrioventricular block; MS = membranous septum; PPM = permanent pacemaker; PPMI = permanent pacemaker implantation; TAVR = transcatheter aortic valve replacement.

Despite recent advances in TAVR, the occurrence of CDs remains a significant complication that requires attention. The predominant mechanism underlying this phenomenon is thought to be the mechanical compression of the atrioventricular conduction system by the transcatheter heart valve.^5^^,^^6^ In particular, transcatheter heart valve placement below the MS is believed to exert direct pressure on the conduction system, leading to CDs. A specific algorithm was established in 2019 to optimize the prevention of sudden cardiac death from post-TAVR CDs and the early discharge of eligible patients.^1^ This algorithm categorizes patients into 3 groups—early discharge, high-risk, and those requiring PPMI—based on pre- and post-TAVR ECG findings to guide postprocedural management. A retrospective validation of this algorithm in a patient cohort with a PPMI rate of 16.3% demonstrated its reliability in determining early discharge eligibility.^4^ The Promote study prospectively evaluated the algorithm in 2,110 patients who underwent TAVR and reported a PPMI rate of 15.6%, thus confirming the algorithm's feasibility and safety.^2^ Although previous consensus algorithms have shown higher NPV for early discharge candidates, stratifying this risk in patients considered at high risk of HAVB or CHB remains challenging. This study highlighted the notion that incorporating MS length assessments in high-risk patients increased the NPV from 88.9% to 95.2%. This suggests that patients with longer MSs, even if initially classified as high risk for HAVB or CHB, may safely undergo early discharge after TAVR.

This retrospective single-center analysis applied the consensus algorithm post hoc; patients were not prospectively managed per algorithm recommendations, limiting causal inference. The decision to perform PPMI was primarily based on the clinical judgment of each physician. PPMI decisions reflected physician judgment and a conservative policy (PPMI for documented CHB or advanced HAVB), which likely lowered absolute PPMI rates compared with multicenter series. We did not assess the pacing dependency rate in our patients who underwent PPMI. Therefore, it remains unclear whether some of these patients could have avoided the need for PPMI.

Among our cohort of patients classified as being at a high risk of experiencing CDs, those with longer MSs were more likely to avoid requiring PPMI. Therefore, incorporating MS length assessment into the 2019 consensus algorithm may improve personalized CD risk stratification and contribute to optimizing post-TAVR management strategies.

## Funding Support and Author Disclosures

Dr Tada is a clinical proctor for Edwards Lifesciences, Medtronic, and Abbott. All other authors have reported that they have no relationships relevant to the contents of this paper to disclose.
